# Importance of patient selection criteria in determining diagnostic copy number variations in patients with multiple congenital anomaly/mental retardation

**DOI:** 10.1186/s13039-019-0436-2

**Published:** 2019-05-27

**Authors:** Şule Altıner, Nüket Yürür Kutlay

**Affiliations:** 1 Department of Medical Genetics, Trabzon Kanuni Training and Research Hospital, University of Health Sciences, Topal Osman Street 7, 61290 Trabzon, Turkey; 2 Department of Medical Genetics, School of Medicine, Ankara University, Ankara, Turkey

**Keywords:** Chromosomal microarray, Multiple congenital anomaly, Mental retardation, Loop hybridization, Patient selection criteria

## Abstract

**Background:**

Etiology of developmental delay/intellectual disability is very heterogeneous. In recent years, genetic causes have been defined through the use of chromosomal microarray analysis as a first step genetic test.

**Results:**

Samples from 30 patients with multiple congenital anomaly and/or mental retardation were analyzed with array comparative genomic hybridization in the context of this study. Before this analysis, karyotyping, subtelomeric fluorescence in situ hybridization and additionally fragment analysis for fragile X in males, had been routinely made all of which were reported to be normal. The purpose of our study was to determine the copy number variations as well as to investigate methods to increase diagnostic yield of array comparative genomic hybridization and forming a suitable flow chart decision pipeline for test indication especially for developing countries. Genomic changes were identified at a rate of about 27% in our series. Although this ratio is higher than the literature data, it could be due to the patient selection criteria.

**Conclusion:**

Chromosomal microarray analysis is not easily utilized for all patients because of its high-cost. Thus, for increasing cost-effectiveness, it may be used step by step for defined targets. Along with discussing the patients with copy number variations relevant with the phenotype, we suggest a flow chart for selection of diagnostic test with the highest diagnostic rate and the lowest expenditure which is quite important for developing countries.

**Electronic supplementary material:**

The online version of this article (10.1186/s13039-019-0436-2) contains supplementary material, which is available to authorized users.

## Introduction

Developmental delay/intellectual disability (DD/ID) which is observed with an incidence of 1–3% in the population is defined as a significant impairment of cognitive and adaptive function before the age of 18. It is a group of disorder associated with personal, familial and social effects [1–3]. Currently, only supportive therapy can be used in this group. Etiology is heterogeneous with many environmental and genetic factors. Genetic alterations such as chromosomal imbalances, known microdeletion/duplication syndromes, gene mutations, environmental factors like teratogens, premature labor, perinatal hypoxia and infections are well defined etiologic factors [4–7]. Despite this variety, etiology is still not defined in all patients [1]. However, due to the complex nature of neural development, to determine all of these factors will take time. Identification of the etiology will contribute to the development of treatment options and to fill the gaps of cognitive development processes. Genetic testing also necessary for prenatal diagnosis of multiple congenital anomaly (MCA) cases where prenatal ultrasonographic diagnosis is not possible.

To reveal the reason of mental retardation will;

- Contribute to disease management,
- Provide early detection and prevention of some pathologic findings (e.g. obesity in Prader - Willi Syndrome),
- Help in acceptance of disability status,
- Provide link between family and support groups,
- Support significant emotional relief in families [8].

Genetic alterations associated with etiology can be shown in 3–5% of cases by high-resolutionG-banding analysis and in 3–6% of the cases by subtelomeric fluorescence in situ hybridization (FISH). Chromosomal microarray analysis is the first step genetic test in this group with a diagnostic yield of 10–20% [1, 9].

Thirty patients with multiple congenital anomaly and/or mental retardation were tested with array comparative genomic hybridization (array CGH) in this study. Before array CGH; conventional karyotyping with at least 550 band-level, subtelomeric FISH and Fragile X analysis in males were performed. Patients who were reported to be normal were selected for the study. In addition to genetic studies, patients with clinical findings that accompany mental retardation and with no defined consanguinity between parents, were selected. In our study, it was aimed to determine the copy number variations (CNVs) that could be associated with the phenotype of the patients. Besides, major aim of the study is to investigate ways to increase the diagnostic yield of array CGH and reduce costs with the correct indications especially in developing countries. With array CGH, genomic changes that could be associated with the phenotypes of patients were identified at a rate of about 27%. Patient selection criteria were considered as the main reason for higher ratio than literature data. Besides, coverage of array CGH platform and reporting criteria could contribute to the result.

Array CGH is a high-cost test. However, the cost decreases when diagnostic yield of the test enhances. We believe that the patient selection criteria used in our study provide cost-effectiveness. There are additional clinical findings related with more than one system accompanying intellectual disability with no parental consanguinity. Besides, with cost-effective methods, such as loop hybridization and increasing platform diversity will create a competition, which may raise test utilization. Although array CGH is adding another 10–20% detection rate of pathogenic genomic imbalances, the underlying cause of the phenotype cannot be determined in almost 50% of patients [10]. In order to increase the diagnostic yield of array CGH, platforms should be altered and diversified in accordance with the discovery of novel disease-causing genes and defined CNVs. Moreover, an increased probe density across exons enables the detection of exonic CNVs in addition to whole gene or multiple exon variation [11]. Thus, for increasing cost-effectiveness, an array CGH analysis platform for defined targets could be used step by step.

## Methods

Thirty patients with multiple congenital anomaly and/or mental retardation (MCA/MR) who were consulted to the Department of Medical Genetics, Ankara University School of Medicine were enrolled in this study.

Ethical committee approval for the study was obtained from Ankara University Medical Faculty Ethical Board on September 22, 2014. Informed consent was obtained from all patients participating in the study.

Every patient underwent a detailed evaluation by medical geneticist, which included prenatal and birth history, pedigree, family history, detailed clinical and dysmorphologic examinations. Step wise approach was followed for genetic tests. High resolution G-banding, subtelomeric FISH and Fragile X fragment analysis in male patients were performed. Besides, work up for a known single gene disorder was done for excluding suspected clinical diagnosis.

The patient selection criteria were defined as follows:

- Normal results of high resolution G-banding analysis, subtelomeric FISH, Fragile X fragment analysis in males, single gene studies (if performed)
- No family history of parental consanguinity.

Peripheral blood specimens were used for all genetic tests mentioned below. A standard protocol was used for conventional karyotyping for both the index patient and his/her parents [12]. Genomic DNA was extracted using MasterPure™ Complete DNA Purification Kit. Fragile X polymerase chain reaction was first performed with Applied Biosystems AmpliTaq Gold 360 DNA Polymerase and 6-carboxyfluorescein (FAM)-labeled FMR1 primers. Challenging samples were also reperformed with Abbott Fragile X PCR set. Gene Scan™-1000 ROX™ was used as size standard product. GeneMapper™Software was used for fragment analysis. Chromosome specific subtelomeric FISH probes (TelVysion Probes, Vysis) were used according to manufacturer’s protocols. Subtelomeric FISH analysis was performed for 41 different subtelomeric specific probes (Additional file 1: Table S1).

Array CGH was performed by using commercially available Oxford Gene Technology (OGT) CytoSure ISCA v2 (4x180k). The platform contains 180,000 probes with 19 Kb spacing in targeted regions and 25 Kb in backbone. Slides were scanned on Agilent microarray scanner. Agilent Feature Extraction Software version 12 was used for processing data. A minimum four consecutive probes were used to call significant CNVs. Anomalies present in less than 30% of the cells are not detectable.

**Fig. 1:**
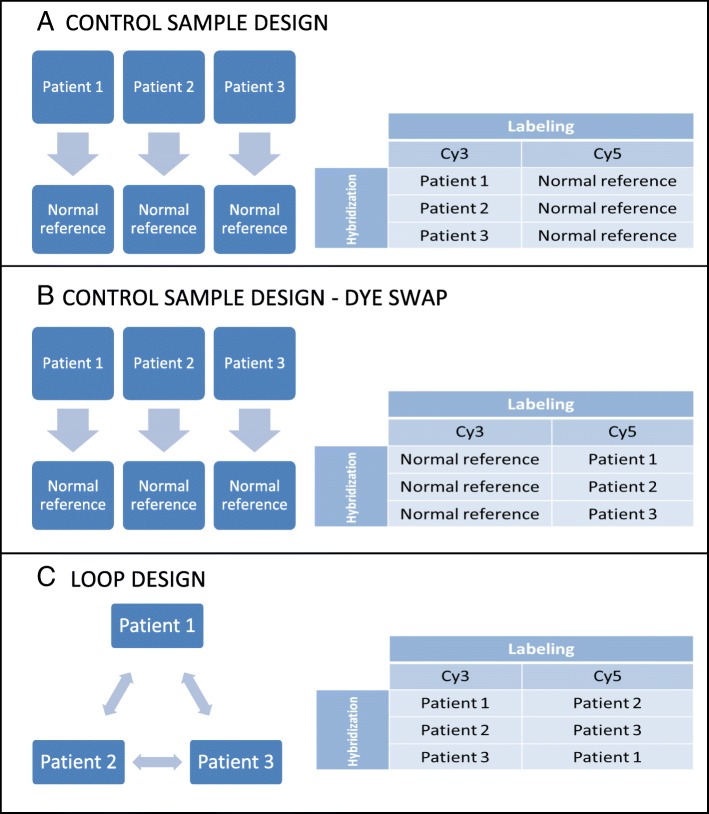
Schematic overview of control sample design (**a**), dye-swap type analysis of control sample design (**b**) and loop design (**c**). Three patients are compared with normal reference in control sample design. Procedure should be repeated via exchanging fluorescent labels of patient and reference. Besides, three patients are compared two by two in loop design (Figure was adapted from Allemeersch et al. [13])

Loop hybridization system was used for 28 patients. Three patients with the same sex but with different phenotypes were selected for the same loop and DNA from one patient is hybridized against DNA from two other patients in this system: patient 1 versus patient 2, patient 2 versus patient 3, and patient 3 versus patient 1 (Fig. 1c). This system measures the intensities of three patient samples in a statistically balanced way and requires no ‘normal reference’ [13, 14]. For two patients, commercially produced ‘normal reference’ was used for hybridization. For doubling, procedure was repeated in a dye swap by exchanging fluorescent labeling of patients and reference samples (Fig. 1 a, b).

Family study was planned in patients with pathogenic CNVs (Case 1, 2, 9, 10, 19, 25, 29 and 30). Only family of Case 29 did not agree to give consent for parental study. Family history was questioned in detail according to the results and suspected siblings were also enrolled in the study in Cases 10 and 19. Affymetrix, CytoScan 750 K SNP microarray platform and Chromosome Analysis Suite (ChAS) software was used for family study of Case 19. Affymetrix, CytoScan Optima SNP microarray platform and Chromosome Analysis Suite (ChAS) software was used for the rest of participants who approved family study.

DECIPHER [15], Database of Genomic Variants [16] and Online Mendelian Inheritance in Man (OMIM) [17] databases were used for interpretation. Results were written according to ISCN 2016 and Genome Reference Consortium human build 37.

CNVs were classified as benign, variant of unknown significance (VOUS) and pathogenic according to gene content and function, inheritance pattern, documentation of multiple peer-reviewed publications [18, 19].

All changes detected over 200 Kb have been reported.

CNVs smaller than 200 Kb were reported if they contain the genes:

- with any genotype-phenotype relation,
- that can be associated with the phenotype of the patient,
- not related with the reason of referral but clinically relevant.

Genetic counseling was given to all patients’ families.

## Results

Our study consisted of 30 patients with MCA/MR, 19 males and 11 females. Of all patients, identified pathogenic copy number variants were detected in 8 (27%). Only one was smaller than 200 Kb (Case 9). Most pathogenic CNVs occurred de novo. No CNVs were detected in one of the cases. Only benign CNVs were detected in four of the cases. VOUS with gene content in 12 patients and CNVs without genes but in reporting criteria due to the size in five, were determined in addition to benign CNVs.

**Fig. 2:**
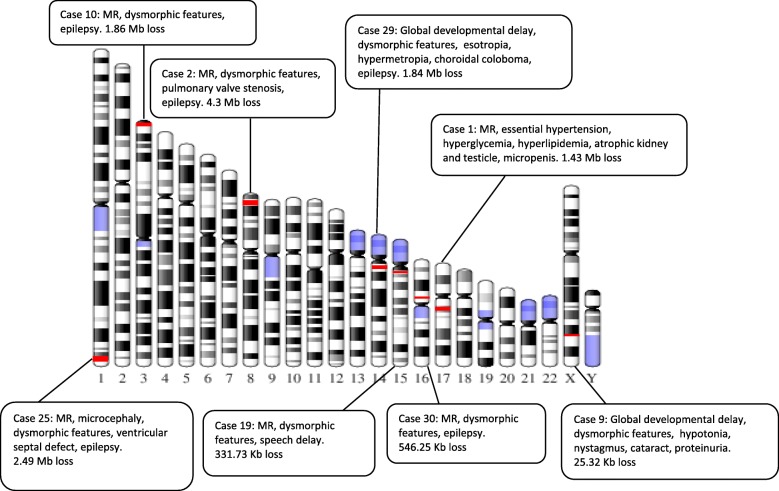
Ideogram presentation of pathogenic CNVs with clinical findings. Red lines represent deletions

Table 1 provides detailed description of patients with pathogenic CNVs. All pathogenic CNVs which ranged between 25.32 Kb to 4.3 Mb in size, are shown in the ideogram together (Fig. 2). Three cases were familial (Case 9, Case 10 and Case 19), four were de novo and one refused consent for parental study (Case 29). Evaluation of phenotypes related with pathogenic CNVs revealed well-defined microdeletion syndromes in three cases (Case 1: 17q12 Microdeletion Syndrome, MIM#614527, Case 25: Chromosome 1q43–44 Deletion Syndrome, MIM#612337, Case 29: Chromosome 14q11-q22 Deletion Syndrome, MIM#613457) [20–24]. Three other cases had well documented microdeletions but penetrance and expressivity of CNVs are known to be variable [Case 10: *CNTN4* deletion in ‘Chromosome 3pter-p25 Deletion Syndrome (MIM#613792)’, Case 19: ‘Chromosome 15q11.2 Deletion Syndrome (MIM#615656)’, Case 30: ‘Chromosome 16p11.2 Deletion Syndrome (MIM#611913)’] [25–29]. Cardiac findings accompanied with mild mental retardation in case 2. The patient was presented with 8p23.1 deletion which covers *GATA4* gene [30]. Chromosome 8p23.1 is described as a critical region for congenital heart diseases. Microcephaly, intrauterine growth retardation, intellectual disability and hyperactive-impulsive behavior are also frequently observed [31]. Case 9 had Lowe Oculocerebrorenal Syndrome (MIM#309000), which is known to be caused by mutations and deletions of the *OCRL* gene [32, 33].

**Table 1:** Clinical and molecular cytogenetic findings in patients with pathogenic CNVs

Case No	Age/ Sex	Clinical findings	Molecular karyotype	Size	MIM disease/OMIM genes	Parental study
1	22/M	Moderate mental retardation, essential hypertension, hyperglycemia, hyperlipidemia, atrophy of left kidney and left testicle, micropenis, Cranial MRI: normal	arr[GRCh37] 17q12(34817554_36249799)×1 dn	1.43 Mb	614,527/ *ZNHIT3, MYO19, PIGW, GGNBP2, DHRS11, MRM1, LHX1, AATF, ACACA, C17orf78, TADA2A, DUSP14, SYNRG, DDX52, HNF1B*	Normal
2	14/M	Small for gestational age at birth, mild mental retardation, high palate, bilateral pes planus and hallux valgus, atrial septal defect, pulmonary valve stenosis, bilateral inguinal hernia and hydrocele, epilepsy, Cranial MRI: normal	arr[GRCh37] 8p23.1(8103647_12404066)×1 dn	4.3 Mb	*CLDN23, MFHAS1, ERI1, PPP1R3B, TNKS, MSRA, PRSS55, RP1L1, C8orf74, SOX7, PINX1, XKR6, MTMR9, SLC35G5, FAM167A, BLK, GATA4, NEIL, FDFT1, CTSB, DEFB136, DEFB135, DEFB134, ZNF705D, USP17L7, USP17L2, FAM86B1, DEFB130, FAM86B2*	Normal
9	1/M	Moderate global developmental delay, frontal bossing, long face, high palate, long filter, short neck, hypotonia, strabismus, nystagmus, bilateral cataract, unilateral cryptorchidism, patent foramen ovale, proteinuria, Cranial MRI: arachnoid cyst	arr[GRCh37] Xq25 (128,671,401 _128696724)×0 mat	25.32 Kb	309,000/*OCRL*	Mother carrier for deletion
10	18/M	Severe mental retardation, epilepsy, synophysis, high nasal root, prognathism, mild pectus excavatum, Cranial MRI: atrophy of cerebral sulcus and fissures	arr[GRCh37] 3p26.3p26.2(1161258_3019093)×1 mat	1.86 Mb	613,792/ *CNTN6, CNTN4*	Mother carrier for deletion
19	7/M	Moderate mental retardation, speech delay, synophysis, large and posterior rotated ear structure, bilateral epicantus, pes planus, Cranial MRI: normal	arr[GRCh37] 15q11.2(22753658_23085387)×1	331.73 Kb	615,656/ *TUPGCP5, CYFIP1, NIPA2, NIPA1*	Mother carrier for deletion
25	8/M	moderate mental retardation, microcephaly, coarse facial appearance, low anterior hairline, bilateral epicantus, upslant palpable fissure, bulbous nose, macroglossia, bilateral single transverse palmar crease, epilepsy, ventricular septal defect, Cranial MRI: Partial callosal agenesis and mild cerebellar atrophy	arr[GRCh37]1q43q44(242854129_245344443)×1 dn	2.49 Mb	612,337/ *CEP170, SDCCAG8, AKT3, ZBTB18, C1orf100, ADSS, C1orf101, DESI2, COX20, HNRNPU-AS1, HNRNPU, KIF26B, EFCAB2*	Normal
29	1/F	Moderate global developmental delay, bilateral strabismus, low set ears, high palate, downturned corners of mouth, micrognathia, bilateral clinodactyly of 4/5 toes, bilateral esotropia, high hypermetropia and isolated choroidal coloboma in the left eye, epilepsy, Cranial MRI: asymmetrical lateral ventricular enlargement	arr[GRCh37]14q11.2(20424745_22263371)× 1	1.84 Mb	613,457/ *PNP, ANG, EDDM3-A,B, RNASE-1,2,3,4,6,7,8,9,10,11,12,13, OR10G-3,2, OR6S1, OR4E1,2, ARHGEF40, SLC39A2, ARHGEF40, SLC39A2, METTL17, NGRG2, TPPP2, ZNF219,TMEM25, HNRNPC, OR5AU1, RPGRIP1, SUPT16H, CHD8, TOX4, RAB2B, METTL3, SALL2*	Not performed
30	20/M	Moderate mental retardation, long eyelashes, prominent maxilla, large ears, retrognathia, epilepsy, Cranial MRI: Perivascular dilatation in the left sublenticular region	arr[GRCh37] 16p11.2(29652360_30198605)×1 dn	546.25 Kb	611,913/ *SPN, QPRT, C16orf54, ZG16, KIF22, PRRT2, MAZ, PAGR1, MVP, CDIPT, SEZ6L2, ASPHD1, TMEM219, KCTD13, TAOK2, HIRIP3, ALDOA, C16orf92, PPP4C, TBX6, INO80E, DOC2A, FAM57B, YPEL3, MAPK3, GDPD3, CORO1A*	Normal

Case 1 had some novel phenotypic findings. The patient had moderate mental retardation, atrophy of left testis and micropenis, also had essential hypertension, hyperglycemia, hyperlipidemia, and atrophy of the left kidney. Although renal anomalies are typically related with 17q12 Microdeletion Syndrome, there are also a few cases with genital anomalies in the literature with similar deletion sizes [20, 21]. There is no reported case of testis atrophy in the literature. Cases with hyperlipidemia have not been reported yet. Hyperlipidemia may be coincidentally present or caused by the syndrome. Besides, mental retardation does not accompany this syndrome. *PIGW* (phosphatidylinositol glycan anchor biosynthesis class w protein) and *LMX1* (Lim homeobox transcription factor 1, alpha) genes could be associated with mental retardation. *PIGW* is an inositol acyl transferase. Compound heterozygous mutations of *PIGW* have been shown in a patient with mental retardation accompanied by hyperphosphatemia and West syndrome. However, this relationship needs to be confirmed with other patients [34]. Although it is not a morbid OMIM gene, *LMX1* is also located in deleted region, may be thought to contribute to mental retardation associated with this syndrome. This gene encodes LIM protein with cystine-rich zinc binding site which plays a role in neuronal differentiation [35].

## Discussion

In this study, genomic changes that could be associated with the phenotype of patients were identified at a rate of about 27% with array CGH. Although this ratio was higher than the literature data, it could be the effect of patient selection criteria. Density and distribution of probes on array platform were considered as other effective factors [9, 10]. Also, our reporting criteria could increase this ratio. According to these criteria, CNVs smaller than 200 Kb were reported if they contained the genes which were probably/potentially associated with the phenotype as in the case 9. Because of big aberrations and unbalanced subtelomeric variations had been detected and excluded with G-banding and subtelomeric FISH analysis respectively, this rate reflects only submicroscopic aberrations except subtelomeric region. In the literature, reported rates include all these anomalies when compared with our results [36, 37]. In addition to this, diagnostic yields of studies without / fewer selection criteria were less when compared to our study. Özyılmaz et al. [38] detected diagnostic yield of microarray as 13.6% among patients with DD, autism spectrum disorders and congenital anomalies. Lee et al. [39] detected diagnostic yield of array CGH as 16.9% among patients with DD/ID and who also had apparently normal karyotype. So we could speculate that our patient selection criteria increased the detection rate of submicroscopic aberrations by array CGH. Hence, step wise approach can reduce the need of using array CGH. Of course, when all test costs are cumulatively evaluated, the final cost is higher than array CGH. But since a lot of patients were diagnosed until this last step, the final cost will be reduced. Although, array CGH is a high-cost test, in cases with additional clinical symptoms of more than one system accompanying intellectual disability, where the parents are non-consanguineous, cost-effectiveness of this analysis rises.

There could be other approaches for increasing the diagnostic yield and rising cost-effectiveness. Instead of subtelomeric FISH, parental conventional cytogenetic analysis could be performed in patients with apparently normal karyotype. This is a more practical and cheaper approach if one of the parents has balanced translocation and patient has cryptic unbalanced subtelomeric rearrangement [40].

All pathogenic cases had deletions in this study. This could be due to the milder phenotypes of patients with duplications and could result in selection bias [2]. Duplications are part of unbalanced translocations in some studies as shown below, which were excluded in our study via subtelomeric FISH analysis. Sharma et al. [36] selected 106 patients with unexplained DD/ID, dysmorphism with or without MCA for microarray analysis. Although, pathogenic CNVs were found in 15 (14.2%) patients, five of them had unbalanced translocations between two subtelomeric chromosomal regions. Eight patients had microdeletions whereas two patients had microduplications. Shoukier et al. [37] applied array CGH for 342 children with unexplained DD/ID. Pathogenic CNVs were detected at 45 (13.2%) patients. Three of them had unbalanced translocation.

Although control sample design is widely used in daily practice, loop design was used in our study. Patient samples are compared to the ‘normal references’ in control sample approach (Fig. 1a). For confirming aberrations, the procedure is repeated via exchanging fluorescent labels of patient and reference samples (Fig. 1b). However, DNA from one patient is hybridized against DNA from two other patients in loop hybridization system (Fig. 1c). In other words, for three patients, three arrays are necessary in control sample design, and three more arrays are used for verifying. Besides, in loop system, three arrays are consumed for three patients but two measures are obtained for each and double analysis is performed for each patient [13]. Analysis of samples were doubled via loop design, which reduced the cost compared to dye swap type control sample design [14].

Loop design is an effective method in patients with congenital anomaly. Likelihood of patients having the same aberrations is low because there are numerous genetic alterations responsible for the etiology of MCA/MR [13]. Patients in the same loop were carefully selected in our study. The patients with similar systemic findings such as hearing loss or obesity were never located at the same loop. All pathogenic CNVs were detected in loop design model and pathogenic CNV detection rate of the study is convincing. As a result, we can say that loop design is also effective method in MCA/MR patients.

Analysis platform had detected a large number of benign CNVs and VOUS. Some VOUS had gene content which little was known about their function. The effect of different CNV combinations is not exactly known. The study of Girirajan et al. [41] is a good example of this subject and it is called ‘two-stroke’ hypothesis of CNVs. In that study, same pathogenic CNVs were compared with phenotypes and genotypes. Presence of a second CNV bigger than 500 Kb was shown significantly in patients with severe phenotypes. Besides, it was suggested that imprinting effect, mutations in regulatory genes and environmental factors may play role in clinical differentiation, as well as non-predictive effects of CNV. Benign or VOUS CNVs can accompany in most of the cases. There is no database for different combinations of these CNVs today. As a result of this, in our study CNVs were classified according to gene content and function, inheritance pattern, documentation of multiple peer-reviewed publications [19]. Each protein coding gene was evaluated with the phenotype of the patient.

## Conclusion

MCA/MR is a heterogeneous group of disorders which requires multidisciplinary and investigational approach to find the etiology. Finding etiology helps disease management, prevents unnecessary tests, offers prenatal diagnosis for future pregnancies, provides early detection and prevention of some pathologic findings, helps acceptance of disability status, provides link between family and support groups and raises significant emotional relief in families [8].

It is not appropriate to assert a certain conclusion because of our small sample size. However, we think that it could lead the way for future studies. Pathogenic CNVs were detected at a rate of about 27% in this study with array CGH. This ratio was higher than literature data, it could be the effect of patient selection criteria.

Narrowing patient selection criteria will increase the diagnostic yield of array CGH. We suggest that the patient selection criteria be as follows:

- Conventional cytogenetic analysis for common aneuploidies (such as trisomy 21, trisomy 18, etc.)
- Conventional cytogenetic analysis for phenotypically normal individuals with familial history of chromosomal imbalance or with recurrent miscarriages
- FISH analysis for well-defined microdeletion syndromes
- Sequencing methods for well-defined single gene disorders

Additionally

- clinical findings of at least two different systems that accompany mental retardation
- no defined consanguinity between the parents

**Fig. 3:**
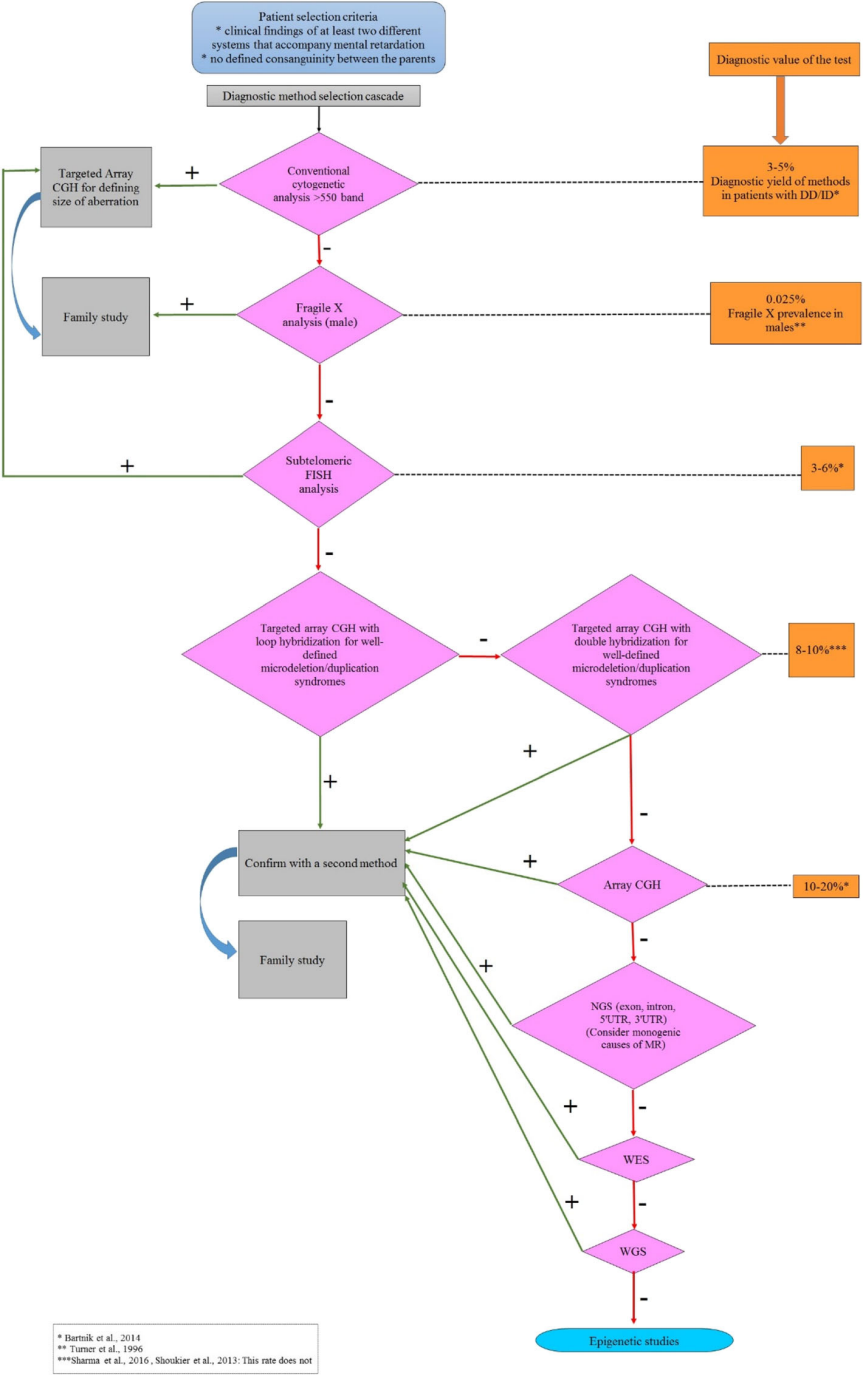
A flow chart for selection of diagnostic test. DD/ID: developmental delay/intellectual disability, NGS: next generation sequencing, UTR: untranslated region, MR: mental retardation, WES: whole exome sequencing, WGS: whole genome sequencing (references used in figure: Bartnik et al. [1], Sharma et al. [36], Shoukier et al. [37], Turner et al. [42])

We suggest array CGH analysis step by step with a flow chart for the selection of diagnostic test especially in developing countries (Fig. 3). According to this flow chart, cumulative diagnostic value of the tests until array CGH is approximately 14–20%. When the diagnostic value of array CGH, which is 10–20%, is taken into account the tests before this step compose greater part of diagnosis.

Besides, databases to reveal the importance of combinations of different CNVs, such as VOUS, benign, should be established in order to interpret the cases more precisely.

Cost of array CGH is still a major concern. We recommend the loop hybridization method for MCA/MR patients. Double analyses of the same patients in loop design reduce the cost compared to dye swap type control sample design.

Despite the development in technology, underlying cause of the phenotype cannot be determined in almost 50% of the patients [10]. In order to increase the diagnostic yield of array CGH, platforms are being altered in accordance with the discovery of novel disease-causing genes. Moreover, an increased probe density across exons enables the detection of exonic CNVs in addition to whole gene or multiple exon variation [11]. Next generation techniques such as whole exome or whole genome sequencing are subsequent choices to determine the etiology of MCA/MR.

## Additional file

Additional file 1: Subtelomeric FISH probes. (DOCX 15 kb)

## Data Availability

Detailed data of the patients reported in this article are available from the corresponding author on reasonable request.

## Abbreviations

array CGH: array comparative genomic hybridization
CNVs: copy number variations
DD/ID: developmental delay/intellectual disability
FISH: fluorescence in situ hybridization
MCA: multiple congenital anomaly
MR: mental retardation
VOUS: variant of unknown significance
