# Supplementary material for: Importance of patient selection criteria in determining diagnostic copy number variations in patients with multiple congenital anomaly/mental retardation
Source: Mol Cytogenet. 2019 May 27;12:23. doi: 10.1186/s13039-019-0436-2 (PMC6537423; doi:10.1186/s13039-019-0436-2)
Supplement: Supplementary file 1 — Subtelomeric FISH probes. (DOCX 15 kb) [file 13039_2019_436_MOESM1_ESM.docx]

| **Additional file 1: Table S1. TelVysion Probes, Vysis** | | | |
| --- | --- | --- | --- |
| **Chromosome** | **Cytogenic Location** | **Probe Name** | **Fluorophore** |
| 1 | CEB108/T7 | TelVysion 1p SpectrumGreen | Green |
| 1 | VIJyRM2123 | TelVysion 1q SpectrumOrange | Orange |
| 2 | VIJyRM2052 | TelVysion 2p SpectrumGreen | Green |
| 2 | VIJyRM2112 (D2S447) | TelVysion 2q SpectrumOrange | Orange |
| 3 | 3PTEL25 (D3S4559) | TelVysion 3p SpectrumGreen | Green |
| 3 | 3QTEL05 (D3S4560) | TelVysion 3q SpectrumOrange | Orange |
| 4 | GS10K2/T7 (D4S3359) | TelVysion 4p SpectrumGreen | Green |
| 4 | AFM A224XH1 (D4S2930) | TelVysion 4q SpectrumOrange | Orange |
| 5 | C84c11/T3 | TelVysion 5p SpectrumGreen | Green |
| 5 | GS35o8/T7 (D5S2907) | TelVysion 5q SpectrumOrange | Orange |
| 6 | 6PTEL48 | TelVysion 6p SpectrumGreen | Green |
| 6 | VIJyRM2158 | TelVysion 6q SpectrumOrange | Orange |
| 7 | VIJyRM2185 | TelVysion 7p SpectrumGreen | Green |
| 7 | VIJyRM2000 | TelVysion 7q SpectrumOrange | Orange |
| 8 | AFM 197XG5 (D8S504) | TelVysion 8p SpectrumGreen | Green |
| 8 | VIJyRM2053 | TelVysion 8q SpectrumOrange | Orange |
| 9 | 305J7-T7 | TelVysion 9p SpectrumGreen | Green |
| 9 | VIJyRM2241 (D9S325) | TelVysion 9q SpectrumOrange | Orange |
| 10 | 10PTEL006 | TelVysion 10p SpectrumGreen | Green |
| 10 | D10S2290 | TelVysion 10q SpectrumOrange | Orange |
| 11 | D11S2071 | TelVysion 11p SpectrumGreen | Green |
| 11 | D11S1037 | TelVysion 11q SpectrumOrange | Orange |
| 12 | 8M16/SP6 | TelVysion 12p SpectrumGreen | Green |
| 12 | VIJyRM2196 | TelVysion 12q SpectrumOrange | Orange |
| 13 | VIJyRM2002 (D13S327) | TelVysion 13q SpectrumOrange | Orange |
| 14 | D14S1420 | TelVysion 14q SpectrumOrange | Orange |
| 15 | WI-5214 (D15S936) | TelVysion 15q SpectrumOrange | Orange |
| 16 | 16PTEL05 | TelVysion 16p SpectrumGreen | Green |
| 16 | 16QTEL013 | TelVysion 16q SpectrumOrange | Orange |
| 17 | 282M16/SP6 | TelVysion 17p SpectrumGreen | Green |
| 17 | D17S928 | TelVysion 17q SpectrumOrange | Orange |
| 18 | VIJyRM2102 (D18S552) | TelVysion 18p SpectrumGreen | Green |
| 18 | VIJyRM2050 | TelVysion 18q SpectrumOrange | Orange |
| 19 | 129F16/SP6 | TelVysion 19p SpectrumGreen | Green |
| 19 | D19S238E | TelVysion 19q SpectrumOrange | Orange |
| 20 | 20PTEL18 (D20S1157) | TelVysion 20p SpectrumGreen | Green |
| 20 | 20QTEL14 | TelVysion 20q SpectrumOrange | Orange |
| 21 | VIJyRM2029 | TelVysion 21q SpectrumOrange | Orange |
| 22 | MS607 | TelVysion 22q SpectrumOrange | Orange |
| X | DXYS129, DXYS153 | TelVysion Xp/Yp SpectrumGreenProbe | Green |
| X | EST Cdy 16c07 for SYBL1 | TelVysion Xq/Yq SpectrumOrange Probe | Orange |
| Y | DXYS129, DXYS153 | TelVysion Xp/Yp SpectrumGreenProbe | Green |
| Y | EST Cdy 16c07 for SYBL1 | TelVysion Xq/Yq SpectrumOrange Probe | Orange |
